# Extractive Bioconversion of Gamma-Cyclodextrin and Recycling of Cyclodextrin Glycosyltransferase in Liquid Biphasic System Using Thermo-Separating Polymer

**DOI:** 10.3389/fchem.2018.00448

**Published:** 2018-10-05

**Authors:** Yu Kiat Lin, Pau Loke Show, Yee Jiun Yap, Arbakariya Ariff, Mohamad Suffian Bin Mohamad Annuar, Oi Ming Lai, Tau Chuan Ling, Eng Poh Ng

**Affiliations:** ^1^Faculty of Science, Institute of Biological Sciences, University of Malaya, Kuala Lumpur, Malaysia; ^2^Department of Chemical and Environmental Engineering, Faculty of Engineering, University of Nottingham Malaysia Campus, Semenyih, Malaysia; ^3^Department of Applied Mathematics, Faculty of Engineering, University of Nottingham Malaysia Campus, Semenyih, Malaysia; ^4^Faculty of Biotechnology and Biomolecular Sciences, Universiti Putra Malaysia, Serdang, Malaysia; ^5^Department of Bioprocess Technology, Faculty of Biotechnology and Bimolecular Sciences, University Putra Malaysia, Serdang, Malaysia; ^6^School of Chemical Sciences, Universiti Sains Malaysia, Gelugor, Malaysia

**Keywords:** liquid biphasic system, ethylene oxide-propylene oxide, extractive bioconversion, cyclodextrin, *Bacillus cereus*

## Abstract

An extractive bioconversion conducted on soluble starch with cyclodextrin glycosyltransferase (CGTase) enzyme in ethylene oxide-propylene oxide (EOPO)/potassium phosphates liquid biphasic system (LBS) to extract gamma-cyclodextrin (γ-CD) was examined. A range of EOPO (with potassium phosphates) molecular weights was screen to investigate the effect of the latter on the partioning efficency of CGTase and γ-CD. The results show that the optimal top phase γ-CD yield (74.4%) was reached in 35.0% (w/w) EOPO 970 and 10.0% (w/w) potassium phosphate with 2.0% (w/w) sodium chloride. A theoretical explanation for the effect of NaCl on γ-CD was also presented. After a 2 h bioconversion process, a total of 0.87 mg/mL concentration of γ-CD was produced in the EOPO/ phosphates LBS top phase. After the extraction of top phase from LBS, four continuous repetitive batches were successfully conducted with relative CGTase activity of 1.00, 0.86, 0.45, and 0.40 respectively.

## Introduction

In this study, liquid biphasic system (LBS) extractive bioconversion was carried out to split the target product and the biocatalyst into top phase and bottom phases, partitioning target biomolecules into one of the phases. These molecules are selectively partitioned based on the surface properties of the molecules and particles such as size, charge, and hydrophobicity (Walter and Johansson, 1994). The LBS extractive bioconversion combined production and recovery technique into a single step. In contrast to conventional processes (e.g., enzymatic bioconversion), the biocatalyst that is retained in one phase is reusable, encouraging a continuous extractive bioconversion process (Charoenlap et al., 2004).

To date, Polymer/polymer LBS is a type of LBS that has been extensively studied and utilized. Efficient and biocompatible polyethylene glycol (PEG)/dextran LBS is the common polymer/polymer LBS that has been widely utilized for the purification and separation of diversified biomolecules such as proteins, nucleic acids, and cell organelles (Lu et al., 1996; Charoenlap et al., 2004). Nevertheless, the application of PEG/dextran LBS in industrial scale has been hampered by the high ocst of the phase-forming chemicals (i.e., dextran) (Lu et al., 1996). Gamma-cyclodextrin's recovery (γ-CD) by LBS was then improved by building a recyclable LBS in which the polymer PEGs were substituted through the use of the copolymer ethylene oxide-propylene oxide (EOPO). The latter is a more economical and environmentally friendly LBS with the ability to maintain the organic activity of the enzyme. EOPO is a copolymer capable of thermos-separating into two phases when the temperature is higher than the lower critical solution temperature (LCST) (Johansson et al., 1997). After heating the system above a certain temperature, the EOPOs split up into two phases, allowing the recovery and reutilization of polymers in subsequent LBS. This novel investigation on the CGTase recovery will bring about a simplification of the CGTase purification steps as well as a reduction in the cost incurred on the environment (Johansson et al., 1999; Dembczynski et al., 2010).

The Cyclodextrins (CDs) are cyclic oligosaccharides that are constructed using cyclodextrin glycosyltransferase (CGTase) enzymes via the transglycosylation process (Rodrigues et al., 2015). CDs have a structural feature enabling them to make up inclusion complexes with an abundance of guest compounds, promoting broad applications of CDs in different industries like pharmaceuticals, food, and chemical products (Martin, 2004). There are three major forms of CDs, namely, α, β, and γ CDs which are formed by six, seven, and eight glucopyranose units respectively (Singh et al., 2002). Among these three types of CDs, the γ-CD has the largest interior cavity and the highest solubility. As shown in the later part of this paper, the size of this interior cavity will, in the presence of the salt, affect the efficiency of the CD extraction, and the large interior cavity size of the γ-CD increases the γ-CD extraction efficiency. However, γ-CD is a lot more expensive than α and β-CD due to its lower production efficiency compared to α and β-CDs (Wang et al., 2007; Moriwaki et al., 2008).

Therefore, an LBS extractive bioconversion is carried in this work to study methods of enhancing the separation and productivity of γ-CD from *Bacillus cereus* CGTase. In addition, approaches of optimizing the γ-CD recovery were studied via the investigation of the effects of selected LBS variables on the γ-CD extraction. These variables include the EOPO molecular weight, tie-line lengths (TLLs), volume ratio and introduction of sodium chloride (NaCl).

## Materials and methods

### Materials

The γ-CD standard, poly (ethylene glycol-ran-propylene glycol) (3,900, 12,000 g/mol) and poly (ethylene glycol-ran-propylene glycol) mono butyl ether (970 g/mol) were bought from Sigma-Aldrich Co. (St. Loius, MO, USA). The potassium phosphate salts (K_2_HPO_4_, KH_2_PO_4_) were bought from Merck (Darmstadt, Germany). Phenolphthalein was sourced from Merck (Darmstadt, Germany). Soluble starch was bought from Becton, Dickinson and company (USA). All other chemicals that were used in this study were of analytic grade.

### *Bacillus cereus* production

The *B. cereus* was cultivated following the process as describe previously (Ng et al., 2011). Culture medium was prepared using 1% (w/v) sago starch, 0.5% (w/v) peptone, 0.5% (w/v) yeast extract, 0.009% (w/v) 0.1% K_2_HPO_4_, 1% Na_2_CO_3_, and MgSO_4_ (autoclaved separately). The inoculum was grown at 37°C for 18 hours (h) with a 250 rpm continuous agitation. The inoculum was then shifted into the CGTase production media, which was thereupon incubated at 37°C for 30 h with a continuous agitation speed of 250 rpm. CGTase was harvested from the supernatant after a 30 min centrifugation at 4,000 rpm.

### Partitioning of CGTase and γ-CDs in LBS

Partition experimentations were executed at room temperature with predetermined amounts of dissolved EOPO, potassium phosphate and distilled water which were mixed in a 15 mL centrifugal tube. 10% (w/w) of crude CGTase and 10% (w/w) of standard γ-CD (50 mg/mL) were then added into the LBSs to attain a total weight of 10 g. The established LBSs were then shaken utilizing vortex mixer and thereupon subjected to a 4,000 rpm centrifugation for 10 min. After the phase separation, bottom and top phases were collected for γ-CD concentrations and CGTase activity analysis.

### CGTase activity analysis

CGTase cyclizing activity was quantified by utilizing the phenolphthalein method (Ng et al., 2011). A 50 μL enzyme sample was added to a 750 μL substrate solutions [1% (w/v) starch in 0.05 M Tris-HCl buffer pH 8.0] which was incubated at 55°C for 10 min. The enzymatic reaction of the CGTase was terminated by introducing 375 μL of 0.15M NaOH, followed by adding 100 μL 0.02% (w/v) phenolphthalein (in 5 mM Na_2_CO_3_) for the spectrophotometrical (550 nm) evaluation of CGTase activity.

### Extractive bioconversion of γ-CD in LBS

Extractive bioconversion of γ-CD was executed at a 50 g reaction total weight in a 250 mL Erlenmeyer flask. The reaction temperature was 55°C. Predetermined dissolved EOPO, potassium phosphates, distilled water, 5% (w/w) soluble starch substrate and 20% (w/w) of the CGTase were added into the LBS to attain a final total weight of 50 g. A control (without LBS phase-forming components) was conducted for soluble starch's enzymatic conversion. Top phases' samples were collected one at a time at regular time intervals and heated in boiling water for 5 min to denature the CGTase. The quantification of the γ-CD concentration was carried out by making use of HPLC instrument.

### Calculation

Relative CGTase activity is defined as the ratio of the enzyme activity (U/mL) to γ-CD concentrations in mg/mL unit.

The volume ratio (VR) is defined as the ratio of top phase volume (V_T_) to the bottom phase volume (V_B_)

(1) $$\begin{array}{l} V_{R}=\frac{V_{T}}{V_{B}}. \end{array}$$

Partition coefficient of CGTase (K_CGTase_) is defined as the ratio of top phase CGTase bioactivity (A_T_) to the bottom phase CGTase bioactivity (A_B_), that is,

(2) $$\begin{array}{l} K_{CGTase}=\frac{A_{T}}{A_{B}}. \end{array}$$

Partition coefficient of γ-CD (K_CD_) is defined as the ratio of top phase γ-CD concentration (C_T_) to bottom phase γ-CD concentration (C_B_)

(3) $$\begin{array}{l} K_{CD}=\frac{C_{T}}{C_{B}}. \end{array}$$

Yield of γ-CD in top phase (Y_T_) is defined as

(4) $$\begin{array}{l} Y_{T}=\frac{1}{1+\left(\frac{1}{V_{R}\times K_{CD}}\right)}\times 100\%. \end{array}$$

## Results and discussion

### Effects of EOPO molecular weight and TLL on Y_T_ of γ-CD and CGTase partitioning

Phase diagrams of different EOPO molecular weight and phosphate salt LBSs were referred to for the evaluation of LBS performance in the Y_T_ of γ-CD and partitioning behavior of CGTase (Show et al., 2000). TLLs were constructed for the CGTase partition experiments and γ-CD. EOPO 970, EOPO 3900, and EOPO 12000 were selected for this study due to their compatibility with biomaterials and capability in developing two phases with the salt components (Persson et al., 2000; Dembczynski et al., 2010).

The highest Y_T_ of γ-CD at 63.1% and CGTase with K_CGTase_ at 3.14 were achieved in EOPO 970/potassium phosphate LBS at TLL of 54.6% (w/w). As seen from the results of Table 1, the highest Y_T_ value was achieved in LBSs comprising of EOPO 970. The EOPO 970 with lower PO content (50%) results in higher Y_T_ of γ-CD in comparison with EOPO with higher PO content (EOPO 3900, EOPO 12000). The lower PO content of EOPOs (EOPO 970) allows maximum solubility of γ-CD in the polymer phase, thereby making the γ-CD precipitation in the interphase avoidable (Huang and Forciniti, 2002). The K_CGTase_ values generally decreased as TLL increased. A possible casue to this would be an additional rise in the concentration of the polymer, resulting in a drop in the free volume of LBS top phase, thereby pulling more CGTase to the LBS's bottom phase (Forciniti et al., 1991). Hence, EOPO 970/potassium phosphate in TLL of 54.6% (w/w) was selected for the following experiments since it exhibited the optimal condition for highest Y_T_ of γ-CD with low K_CGTase_.

**Table 1:** Effect of the EOPO molecular weight and TLL upon the K_CGTase_ and Y_T_ (%).

**EOPO molecular weight (g/mol)**	**TLL (%,w/w)**	**K_CGTase_**	**Y_T_ (%)**
970	52.3	5.16	47.6
	54.6	3.14	63.1
	58.0	4.20	58.4
	61.8	4.06	53.0
3,900	36.4	8.44	46.5
	41.2	8.69	46.3
	44.6	6.06	56.4
	48.5	4.19	55.9
12,000	31.6	3.74	52.1
	34.4	3.65	54.7
	42.5	2.79	51.6
	50.2	2.04	47.5

*The data obtained was presented as the average of approximated triplicate readings with an accuracy of ±5%*.

### Effect of V_R_ on the Y_T_ of γ-CD and partitioning of CGTase

**Figure 1:**
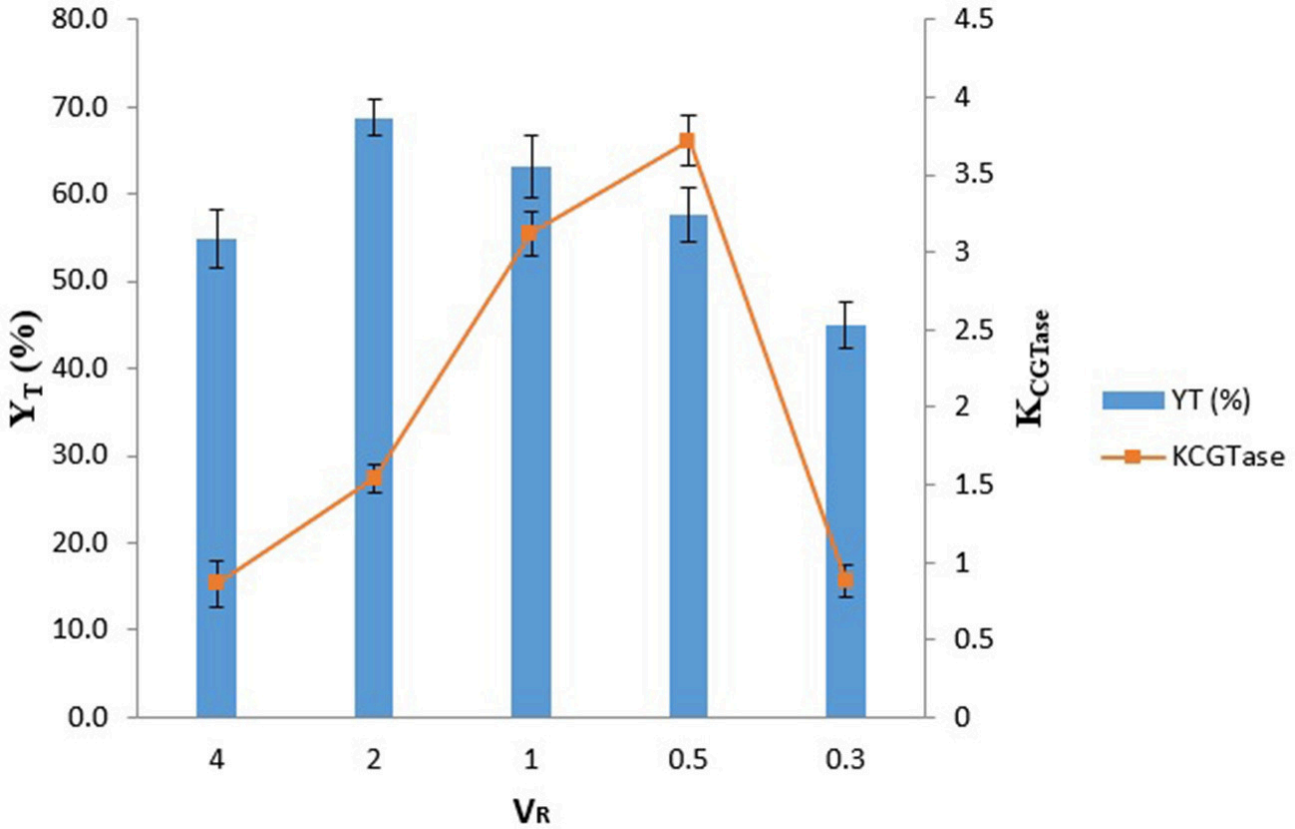
Influence of V_R_ on the partitioning of CGTase. The data obtained was presented as the average of triplicate readings.

The K_CGTase_ and Y_T_ at different V_R_ (V_R_ = 0.3 to 4.0) for EOPO 970/potassium phosphate TLL 54.6% (w/w) were illustrated in Figure 1. The highest Y_T_ of γ-CD (Y_T_ = 68.7%) was reached at V_R_ 2.0. The Y_T_ was generally increasing as the V_R_ increased. This may be due to the fact that a higher V_R_ (i.e., V_R_ > l) resulted in more free volume in the top phase, thereby increasing the solubility limit of Y_T_, which in-turn caused more γ-CD to be attracted to the top phase (Schmidt et al., 1994). In the system of V_R_ 0.5, it is noticeable that V_R_ 05 was given the highest K_CGTase_ which was around 3.5. This is because less free volume in the top phase which give indirectly stress the K_CGTase_ increase. Since in this stage, we are aimed to get the highest Y_T_ of γ-CD instead of high K_CGTase._ Therefore, the optimal condition (V_R_ = 2.0) was selected for subsequent studies.

### Effect of NACL on Y_T_ of γ-CD

The EOPO 970/potassium phosphate in TLL 54.6% (w/w) with VR 2.0 was used to examine the effect of adding NaCl, ranging from 0% (w/w) to 4% (w/w), on the Y_T_ of γ-CD. The partitioning behavior of γ-CD in LBS was due to the difference in hydrophobicity between the phases caused by the addition of NaCl. In addition, the electrostatic potential created by the NaCl contributed to the driving of the γ-CD to the top phase, thereby proliferating the top phase partitioning of γ-CD (Zaslavsky et al., 1991). The interaction between the hydrophobic chain of the EOPO and hydrophobic surface of the γ-CD was enhanced by the addition of the salts. This enhancement was precipitated by the effect of the salt on the hydrophobic interactions and the water solvent structure. The target biomolecules would be thus partitioned to the polymer rich top phase (Albertsson, 1986). Just like the case of PEG-salt system mentioned in Huddleston et al. (1991), when the NaCl concentration is lower, the electrostatic field due to the Na^+^ and Cl^−^ ions will play a greater role in the γ-CD extraction. However, a high NaCl concentration causes a lot of γ-CD to partition to the top phase. This large number of γ-CD molecules bind with the EOPO polymers through hydrophobic interactions, thereby forming larger hydrophobes and repelling the water molecules to the bottom phase. With a reduction in the water molecules in the top phase, there is less water for the γ-CD molecules to dissolve in, thereby causing a reduction in the yield of the γ-CD in the top phase. Thus, although the NaCl serves to help increase the yield of γ-CD, too high a salt level will have the reverse effect of reducing the yield. From Table 2, it can be seen that an optimal yield of γ-CD occurs at an NaCl concentration of roughly 2%(w/w).

**Table 2:** Influence of the NaCl concentration on Y_T_ (%).

**NaCl concentration %(w/w)**	**Y_T_ (%)**
0	68.3
1	71.7
2	74.4
3	72.8
4	72.1

*The data obtained was presented as the average of approximated triplicate readings with an accuracy of ±5%*.

The effect *V*_*E*_ of the electric field (Lemeshko et al., 2013) on the molecules is given as

(5) $$V_{E}=-\underline{E}\cdot \underset{n}{\sum^{\text{​}}}q_{n}rn$$

where is the electric field, *q*_*n*_ is the charge of electron *n*, _*n*_ is the position vector of electron *n*. By approximating the perimeter of a γ-CD molecule to a circle with the origin of at the center of the circle and taking the charge of an electron to be a constant *k*_*e*_ (i.e., the Coulomb constant), we can denote the radius of the γ-CD molecule as *R*, and Equation (5) can be rewritten as

(6) $$\begin{array}{l} V_{E}=-\underline{E}\cdot \sum_{n}q_{n}r_{n}{\underline{\hat{r}}}_{n}=-\underline{E}\cdot \sum_{n}k_{e}R{\underline{\hat{r}}}_{n}=-k_{e}R\sum_{n}\underline{E}.{\underline{\hat{r}}}_{n}\\ \;=-k_{e}R\sum_{0}^{\frac{\pi }{2}}\left|\underline{E}\right|\cos\theta =-k_{e}RA\left|\underline{E}\right| \end{array}$$

where θ is the angle between and _*n*_ and $A=\overset{\frac{\pi }{2}}{\underset{0}{\sum }}\cos\theta$ = constant. Thus, from Equation (6), we can see that the higher the NaCl concentration, the larger the electric field, and hence the larger the effect on the molecule. Additionally, we will expect the γ-CD, which has a larger *R* then α-CD and β-CD, to be more affected by the presence of NaCl in terms of the electric field created.

**Figure 2:**
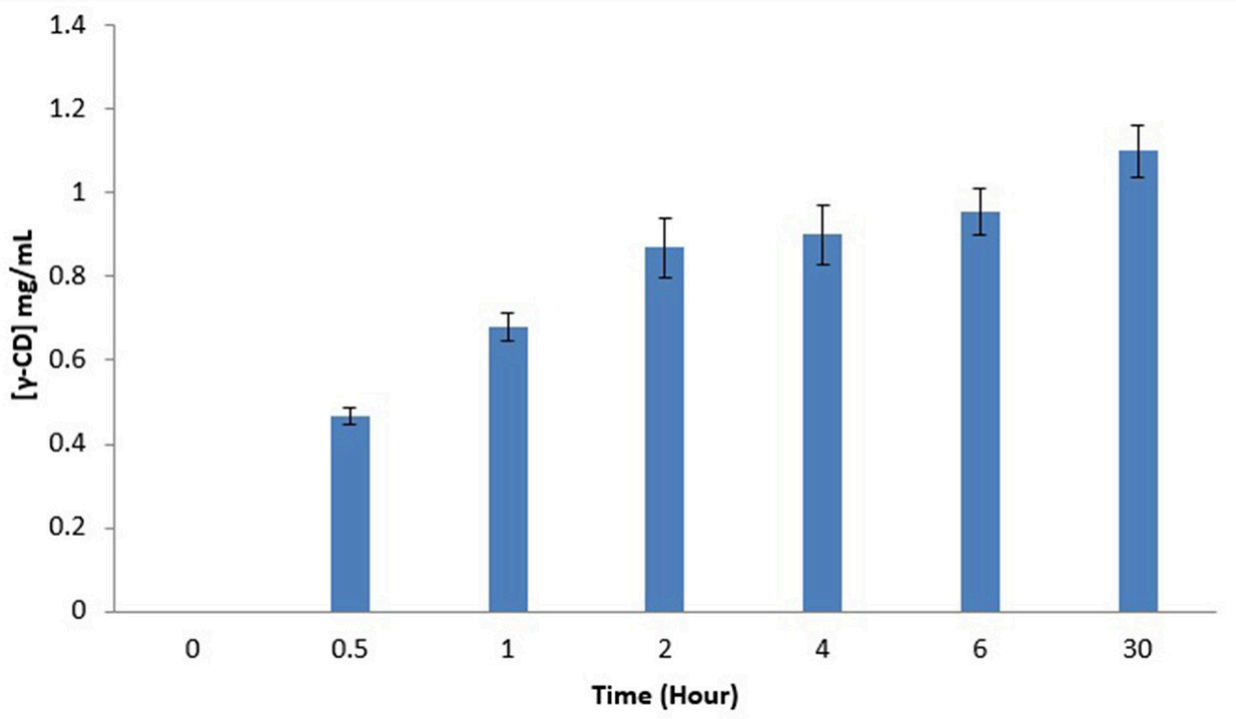
Effects of LBS extractive bioconversion on γ-CD production over time. The data obtained was presented as the average of triplicate readings.

Figure 2 illustrates γ-CD concentration in LBS top phase vs. the extractive bioconversion process time. A 50 mL of EOPO 970/potassium phosphate at TLL of 54.6% (w/w) with VR of 2.0 was employed in this study. 20% (w/w) crude CGTase and 5% (w/w) of soluble starch were added to the LBS. A 1.10 mg/mL concentration of γ-CD was collected in the LBS top phase sample after a 30 h extractive bioconversion process. Nevertheless, 2 h (0.87 mg/mL) is proposed as the harvest time since increase in the γ-CD concentration is minimal after 2 h. A comparison of the CD concentration ratios (for different CDs) between the control sample and the 2 h LBS top phase sample is shown in Table 3. The γ-CD concentration ratio of the top phase sample (17.5%) is much higher than that of the control sample (6.6%). This implies that a higher γ-CD ratio product is achievable by using the LBS extractive bioconversion process.

**Table 3:** A comparison of CDs concentration ratio (%) between LBS top phase sample and control sample.

**CD type**	**Control (%)**	**2 h LBS Top phase (%) (This work)**	**1 h LBS Top phase (%) (Our previous work, Lin et al., 2015)**
α	67.1	59.0	55.4
β	26.3	23.5	25.6
γ	6.6	17.5	19.0
Total	100.0	100.0	100.00

*The data obtained was presented as the average of approximated triplicate readings with an accuracy of ±5%*.

### Repetitive batch for γ-CD production and comparative study by using PEG

**Figure 3:**
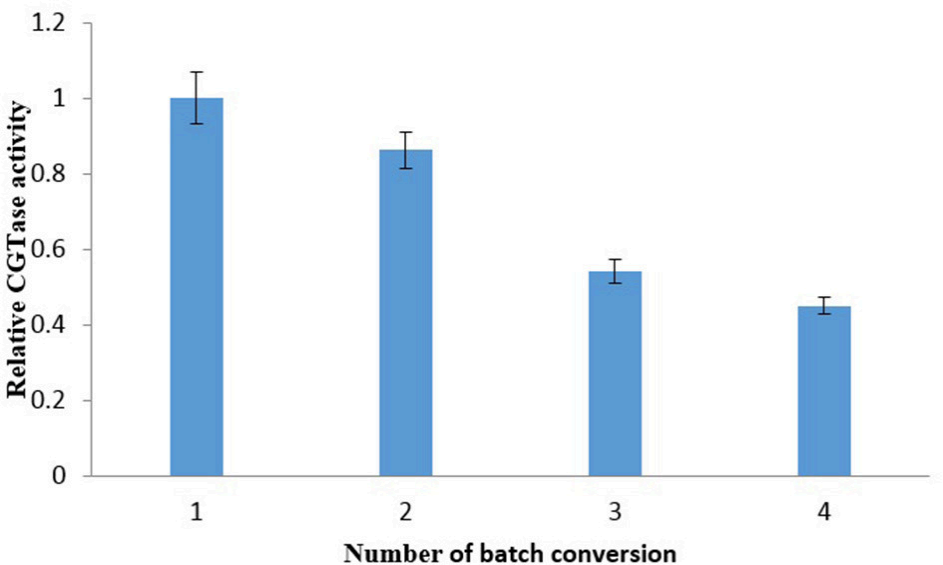
Relative CGTase activity in each batch of soluble starch bioconversion by recycling of the phase components and CGTase. The data obtained was presented as the average of triplicate readings.

LBS's repetitive batch investigation was conducted to study the recycling of the CGTase enzyme in the bottom phase. The repetitive batch of the γ-CD recovery was carried out through EOPO 970/potassium phosphate LBS at 54.6% (w/w) TLL, V_R_ of 2.0, added with 20% (w/w) of crude CGTase, and 5% (w/w) of soluble starch. Top phase extractions were done at a regular interval of 2 h extractive bioconversion process. Figure 3 shows CGTase activity of the bottom phase after extraction of the top phase, and it can be seen that the CGTase activity continues to drop with subsequent batches. The relative CGTase activity went down to 0.45 in the 4th batch of extractive bioconversion. This outcome revealed that the losing of CGTase occurred when the top phase, which contained a certain amount of CGTase (Tramice et al., 2008), was removed. Therefore, it can be seen that to ensure the effectiveness and performance of bioconversion, the CGTase enzyme in the bottom phase should not be recycled more than once. One of our earlier studies about extractive bioconversion of γ-CD from soluble starch with CGTase enzyme by using PEG instead of EOPO (Lin et al., 2015). Table 3 shows the γ-CD concentration ratio within 2 h in the present study was lower than that within 1 h in our previous study (Lin et al., 2015). However, the LBS using EOPO is more economical and environmentally-friendly than that using PEG, as described in introduction. The comparative studies were shown in Table 3.

## Conclusions

In this study, EOPO polymer has been applied to the LBS extractive bioconversion of γ-CD. Generally, the partitioning of γ-CD in EOPO/potassium phosphate LBS was influenced by the PO content and concentration of the EOPO used. The optimal extractive bioconversion of γ-CD in top phase of the LBS was reached in EOPO 970/potassium phosphate LBS of 54.6% (w/w) TLL, V_R_ of 2.0. A γ-CD concentration of 0.87 mg/mL with a 17.5% concentration ratio was retrieved after a 2 h extractive bioconversion process. The objective of this study, which is to produce and recover γ-CD by directly using LBS, has been achieved. In particular, it has been shown that the EOPO 970/potassium phosphate LBS extractive bioconversion is a better alternative technique for producing the γ-CD compared to the conventional method as this alternative technique merges γ-CD production and purification into a single-step process.

## Author contributions

YL performed the experiment and data analysis as well as wrote the manuscript. PS conceived and designed the experiment. YY provided theoretical explanation and analysis for the experimental data. AA, MA, OL, TL, and EN revised the manuscript.

### Conflict of interest statement

The authors declare that the submitted work was not carried out in the presence of any personal, professional or financial relationships that could potentially be construed as a conflict of interest.
